# Emerging Fungal Infections in the Era of Antibiotic Stewardship

**DOI:** 10.1155/2019/5963143

**Published:** 2019-06-02

**Authors:** Justina Truong, Raquel Vertees, Tyson Dietrich, Jeffrey Maguire-Rodriguez, John Ashurst

**Affiliations:** ^1^Kingman Regional Medical Center, Department of Emergency Medicine, 3269 Stockton Hill Road, Kingman, AZ 86409, USA; ^2^Kingman Regional Medical Center, Department of Pharmacy, 3269 Stockton Hill Road, Kingman, AZ 86409, USA; ^3^Kingman Regional Medical Center, Department of Internal Medicine, 3269 Stockton Hill Road, Kingman, AZ 86409, USA

## Abstract

Pneumonia is one of the most common causes of infection seen worldwide and still remains one of the most common causes of mortality despite significant advancements in medicine. With the increase in immunosuppression and antimicrobial usage, emerging infectious agents have been isolated in patients with pneumonia. The authors present a case in which *Nonomuraea solani*, *Candida glabrata*, and *Candida dubliniensis* were isolated from a bronchoalveolar lavage from an immunosuppressed patient with pneumonia.

## 1. Introduction

The American Thoracic Society defines dyspnea as a subjective experience of breathing discomfort that is composed of qualitatively distinct sensations that vary in intensity [1]. Dyspnea accounts for between 3 and 4 million emergency department visits annually, and pneumonia has been attributed as one of the most common causes [2–4]. In the United States, pneumonia is the third leading reason for hospital admission and the number one leading cause of sepsis and death due to infection [5, 6]. As the morbidity and mortality rate due to pneumonia continues to remain stable despite advances in medicine, the number of likely causative agents of infection is increasing [6].

The issue of emerging infectious diseases is a growing concern across the globe [7]. Many etiologic, environmental, and demographic factors contribute to the risk of developing an infection caused by a rare pathogen [7]. The evolution of viral and microbial variants and selection for drug resistance also increase the risk for disease emergence [7]. Example risk factors include older age, chronic lung diseases, immunosuppression due to chronic diseases, or medication use such as glucocorticoids [8]. The authors present a case of pneumonia in a patient with many of the aforementioned risk factors who had a bronchoalveolar lavage positive for *Nonomuraea solani*, *Candida glabrata*, and *Candida dubliniensis*.

## 2. Case Report

A 64-year-old female presented to the emergency department secondary to shortness of breath, cough, and associated fever. She had a past history of chronic obstructive pulmonary disease, left upper lobe cavitary lung lesion, and microcytic anemia. The patient was on daily oral steroids for the last several years due to poorly controlled COPD and had recently been released from the hospital two weeks before for a left lower lobe pneumonia. At the time of her prior admission, she was started on vancomycin and aztreonam for her pneumonia and was ultimately discharged on levofloxacin for ten days.

During her emergency department course, her chest radiograph revealed a worsening left lower lobe infiltrate which was later confirmed on computed tomography of the chest. The patient had worsening hypoxia during her course and was eventually placed on BIPAP therapy, and she was started on intravenous vancomycin, levofloxacin, and fluconazole for a presumed hospital-acquired or fungal pneumonia given her recent hospital admission and cavitary lung lesion. Prior to admission, she had no physical exam findings to suggest a fungal infection. She was admitted to the hospitalist service for further evaluation and management.

While hospitalized, the patient continued antibiotic and antifungal therapy, and on day two, aztreonam was added due to a worsening clinical picture. The following day, the patient underwent consultations from infectious disease, pulmonology, and cardiothoracic surgery due to her worsening clinical status and pneumonia with associated cavitary lung lesion. Following consultations, the patient underwent a fiberoptic flexible bronchoscopy with bronchoalveolar lavage which showed a large mucous plug obstructing the left main bronchus but no associated lesions. Cultures from the bronchoalveolar lavage eventually grew *Nonomuraea solani*, *Candida glabrata*, and *Candida dubliniensis*.

Following a protracted hospital course of nine days, the patient was discharged home with cefpodoxime 400 mg twice a day for ten more days and was instructed to follow up with infectious disease within the next two weeks. However, the patient eventually presented to the emergency department one month later with a left-sided empyema status after wedge resection of the cavitary lesion that grew *Corynebacterium amycolatum* and *Staphylococcus hominis* spp. on cultures.

## 3. Discussion

Although the most common bacterial cause of pneumonia is still *Streptococcus pneumoniae,* emerging bacterial, fungal, and viral agents have been identified at an alarming rate across the globe. Immunosuppression and an increase in antimicrobial usage appear to play a major role in the development of these new infectious agents. Our patient was chronically immunosuppressed and had been given antibiotics for numerous infections over the last year. Based upon culture results from our patient, three rare organisms were isolated and thought to have played a role in causing her pneumonia and will be discussed.

The Gram-positive, aerobic actinomycete *Nonomuraea solani* was first identified and isolated from an eggplant root in 2013 [9]. Until now, most of the 38 species of *Nonomuraea* have been found almost exclusively in soils and sediments from aquatic and terrestrial habitats and have never previously been isolated from human cultures [10, 11]. Infections with respiratory actinomyces have been identified and present with nonspecific symptoms similar to most other pulmonary infections [12]. Definitive diagnosis of actinomyces and therefore *Nonomuraea solani* can be difficult despite chest radiography, computed tomography, and bronchoalveolar lavage. Treatment of actinomycetes includes beta-lactams, with penicillin G or amoxicillin being the drug of choice [11]. Prolonged, high-dose therapy with beta-lactam antibiotics is recommended for treatment in those with chronic lung disease or other immunosuppression [12].

Another rare species with increasing emergence as a human pathogen is the fungus *Candida glabrata.* Although *Candida albicans* is the main cause of candidosis, *C. glabrata*, a non-*Candida albicans Candida* (NCAC) species, is responsible for an increasing amount of human fungal infections and carries a relatively high morbidity and mortality [13, 14]. Many factors may attribute to its pathogenicity including the ability to form biofilms and adhere to and survive on a wide range of surfaces longer than most *Candida* species. *Candida* species including *C. glabrata* are most commonly found in the oral cavity and vulvovaginal tracts, and overgrowth can be commonly seen in certain instances [13]. For example, host risk factors such as extended hospitalization, immunosuppression, and repeated prior antimicrobial use play a role in disturbance of normal flora [13, 14]. A result is the increased rate of opportunistic infections caused by *C. glabrata*, and cases of pneumonia have even been reported [13, 14]. Moreover, *C. glabrata* has a high resistance to traditional antifungal therapies [14]. Treatment options are somewhat limited for fungal infections, especially those with high resistance to standard azole therapy. Due to its high resistance to azole therapy, amphotericin B has been considered the gold standard for treatment. More studies are needed to understand the virulence and resistance of *C. glabrata* in order to develop effective pharmacological treatment therapies in the future [13, 14].

*Candida dubliniensis* is yet another non-*Candida albicans Candida* (NCAC) species responsible for emerging infections in human hosts [15]. As with the previously discussed *C. glabrata*, patients at risk for *C. dubliniensis* infection include those who are immunosuppressed and those with prolonged or repeated antibiotic courses [15]. Reportedly less virulent than *C. albicans*, it is somewhat unclear what contributes to this species true infectious nature, but there are reports of patients suffering from pneumonia caused by *C. dubliniensis* [15, 16]. Diagnosis and treatment of *C. dubliniensis* is similar to that of all *Candida* species. A wide variety of testing is available, and it is difficult but important to distinguish *C. dubliniensis* from other *Candida* species due to its resistance patterns [17]. Studies have shown that *C. dubliniensis* isolates were susceptible to a wide range of antifungals including voriconazole and amphotericin B, but some isolates were resistant to fluconazole [16].

## 4. Conclusion

The importance of appropriate antimicrobial use has been highlighted by emergence of resistance organisms. Although resistance is a major focus of antimicrobial issues, use can also elicit infections caused by organisms not commonly seen in every day practice. This case highlights why antimicrobial stewardship remains a key factor in not only combating the emergence and reduction of resistant organisms but also impacting the occurrence of infections caused by otherwise nontypical pathogenic organisms.
